# HBV Induced HCC: Major Risk Factors from Genetic to Molecular Level

**DOI:** 10.1155/2013/810461

**Published:** 2013-08-07

**Authors:** Ambreen Ayub, Usman Ali Ashfaq, Asma Haque

**Affiliations:** Department of Bioinformatics and Biotechnology, Government College University Faisalabad (GCUF), Faisalabad 38000, Pakistan

## Abstract

Hepatocellular carcinoma (HCC) is a deadly and emerging disease leading to death in Asian countries. High hepatitis B virus (HBV) load and chronic hepatitis B (CHB) infection increase the risk of developing HCC. HBV is a DNA virus that can integrate DNA into host genome thereby increase the yield of transactivator protein HBxAg that may deregulate many pathways involving in metabolism of cells. Several monogenic and polygenic risk factors are also involved in HCC development. This review summarizes the mechanism involved in HCC development and discusses some promising therapies to make HCC curative.

## 1. Introduction

HBV infection is a major health problem leading to significant death rate worldwide, especially in developing countries including Pakistan. HBV infects 350 million people worldwide, and 7–9 million in Pakistan [1]. HBV is a small enveloped DNA virus pertained to the hepadna family of viruses that integrates its DNA into the host genome and this integration of DNA is believed, in part, to be carcinogenic. Currently at least 10 HBV genotypes and several subtypes have been identified. Well-known genotypes are eight named as A to H [1]. The prevalence of specific genotypes varies geographically; genotype B is primarily found in south Asia with a unique genome structure [2] it was estimated that there is about 8% or <8% complete nucleotide sequence divergence in these genotypes [3, 4]. No differences in viral loads were found in relation to age, gender, or genotype in the African black HCC patients, whereas recent studies from Taiwan reported that HCC patients younger than 40 years of age had lower HBV DNA titre than older patients [5, 6]. HBV contains four overlapping reading frames (ORF): S, P, X and pre C. The SORF encodes the three viral surface proteins: the preS1 (or Large), the preS2 (or Middle) and the S (or small) that corresponds to HBsAg. The soluble antigen “e” (HBeAg) and (HBcAg) encoded by pre-C ORF. The viral polymerases possess DNA polymerase, reverse transcriptase and RNase H activities, and terminal proteins are encoded by P ORF. The X ORF encodes the regulatory X protein, which is capable of transactivating the expression of numerous cellular and viral genes and vital for virus replication [7]. The HBsAg is the first serological marker detectable in serum, primarily appears during the incubation period of virus life cycle and rapidly rises in titer. Another core antigen HBeAg was reported to inhibit production of interleukin 6 (IL-6) through the suppression of nuclear factor kappa B (NF-*κ*B) gene expression. It has been demonstrated that HBeAg could impair both adaptive and innate immune response in order to promote HBV infection. One study reported that the inflammatory cytokines such as IL (12A, 8, 6), IFN-*α*1 and *β*, and TNF mRNA expression downregulated in HBeAg-positive hepatic cell. Thus, it can be concluded that HBeAg can modify the gene expression of tumor suppresser genes in HBV infected patients [8]. HBcAg is a sign of active viral replication. It has been found that agglomeration of naturallyoccurring mutations in particular segments of HBcAg may be related to the development of HCC. Other patent risk factors for HCC include chronic viral hepatitis, cirrhosis, nonalcoholic fatty liver disease, heavy alcoholism and some particular inherited metabolic conditions such as alpha-1-antitrypsin deficiency and hemochromatosis [9], oxidative stress, aflatoxin B1, drugs, medications, chemicals, and up/downregulation of several cellular and immune responsive genes, and so forth. Expression analysis done by tissue microarray technique shows that if the expression of HBsAg, HBcAg, p21 and Rb proteins in HCCs is downregulated, the possibilities of HCC development will increases as these proteins are also tumor suppressors [10]. The present review will focus on the genetic causes of HCC; genes involve in the development of HCC and the role of HBx protein. Although vaccination available but once a person got infected with HBV, there is no treatment other than the supportive care.

## 2. Hepatitis Virus Genome Organization

The HBV genome has many features that distinguish it from other viral genomes for example, (a) circular DNA conformation (b) partially double-stranded DNA and (c) dependence on a reverse transcriptase. It has been concluded that dependence on reverse transcriptase helps in the persistence of viral infection in host hepatocytes. Genome size of HBV is about 3.2 kb having four open reading frames (ORF): S, C, P, and X. The ORFS encodes the viral envelope proteins (HBsAg) and has been divided into three main parts preS1, preS2, and S on the basis of structure and functions. The *C* gene encodes HBcAg and HBeAg. The C gene encodes a core and precore region. The product is HBeAg; if the translation begins from the precore region. If translation starts from core region then the product is HBcAg. The function of HBeAg needs more attention to get fully understood although most of the literature depicts that HBeAg promotes viral persistence in hepatocytes [11]. The largest protein of HBV is P, which encodes for DNA polymerase. The P ORF has been divided into three domains: (1) the reverse transcriptase (RT) domain (2) ribonuclease H domain and (3) terminal protein domain. The function of terminal protein domain is to initiate of minus strand synthesis and encapsidation. The HBV X ORF HBxAg whose main function is in carcinogenesis. Other functions of X gene are repairing of DNA, signal transduction, transcriptional gene activation and to stop protein degradation [12–14], and so forth. Human are natural host of HBV.

## 3. HBV Integrated DNA and Gene in Host Genome Causes HCCs

HBV DNA integration into host genome is a compelling step during CHB infection. Viral DNA integration disrupts the functioning of several genes which are important for normal cell growth and differentiation. The chance to get HCC by HBV is directly proportional to the number of random integration of viral genome in to host liver cells [15]. The integration of HBV DNA into hepatocytes is an integral step for persistent viral infection that leads to CHB infection, which ultimately causes HCC [16]. As viral DNA integration rearranges both host and viral genes leading to the production of altered protein products making hepatocytes more susceptible [17]. The insertion of viral genome results in chromosome deletions and other general genomic instability [18] that may activates several pathways switching on HCC development [19]. Studies revealed that HBx, hepatitis B spliced protein (HBSP) and truncated preS2/S gene, found more frequently than other genes in infected cells. It was demonstrated that expression of HBV proteins have a direct effect on many cellular functions, and some of these gene products can promote malignant transformation in hepatocytes [20, 21]. It was studied that the prevalence of pre-S deletions was significantly higher in HCC patients [22]. It was also suggested in 2010 that there is a strong link between pre-S2 deletion and HCC development [23]. The truncated pre-S2/S of HBV virus induces increased cell proliferation and strong endoplasmic reticulum stress, which induces oxidative stress and DNA damage; ultimately leading to HCC development [24, 25]. The HBSP has been found more frequently as compare to other proteins in HBV infected patients. HBSP may account for the association with HCC [26]. HBSP has found to be involved in persistence of HBV infection, this function of HSBP should be count as one of the dominating cause for HCC [27]. 

## 4. Role of HBx in HCC Development

Hepatitis B virus X (HBx) gene plays a central role in HBV-related HCC progression and stimulation [28]. By promoting the level of G1 protein, HBx protein affects the normal physiology of hepatocytes and cell cycle progression. It was studied that G1-promoting proteins acts as activators for CDK4, requires for HBV replication [29]. Previous studies suggest that up to 50 thousand copies per cell [30]. Quiescent cells may also undergoes cell cycle progression through the expression of HBx protein in infected cells [31]. Moreover, HBx act as an activator for a huge range of viral promotors [32–34]. Thus HBx gene expression is of prime importance for viral reproduction within living cells [35]. Most studies demonstrated that HBx protein mainly expressed in cytoplasm but may also be detectable in nucleus of infected cells [36–42]. It was disclosed by Chen and his colleagues that HBx protein through a Crm-1-dependent nuclear export pathway shuttled between the cytoplasm and nucleus. Hbx protein enhances NF-*κ*B localization to the nucleus, with subsequent activation of respective transcriptional pathways [43]. This ultimately results in the progression of HCC development.

## 5. Impact of HBx on the Regulation of Signaling Pathways

A group of scientists in Germany indicate that HBx is fundamentally present in the cytoplasm and stimulates protein kinase C and other proteins that are activated by stress stimulation, that is, Jun N-terminal kinase, Inhibitor Kappa B Kinase (IKK), Janus kinase/STAT, Phosphoinositide 3-kinase (PI-3-K) and protein kinase B/Akt. HBx protein can also be detectable in the power house of infected cells, where it increases the expression of B-cell lymphoma-2 (Bcl-2) family [44] and increase the risk of HCC formation. Different studies indicate that HBx protein works in cooperation with cellular transcription factors and induces transformation [45] in liver cells causing tumors [46, 47] or cancer [48–50]. Thus, Studies proved that expression of HBV X gene in hepatocytes is a positive hallmark of HCC. It was reported that bone morphogenetic protein (BMP-9) plays a crucial role in nonhepatic tumors [51]. Whereas BMP4 and BMP7 were up-regulated in cirrhosis, BMP4 and BMP7 along with SRC were further up-regulated in hepatocellular carcinoma [52]. Thus, BMP is related to invasiveness of HCC (Table 1).

## 6. HBx and Transcription Regulation

**Figure 1:**
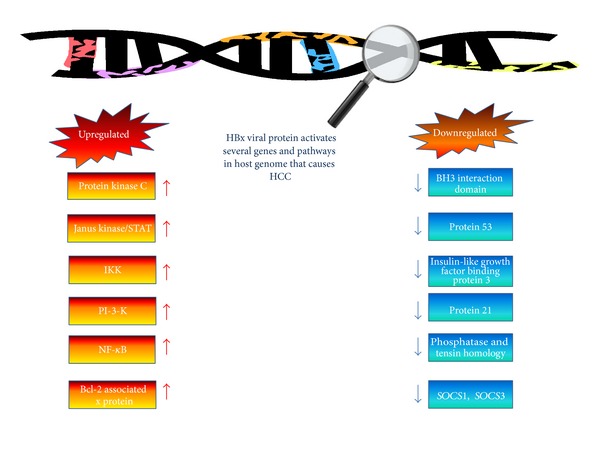
HBx protein activated Pathways and Genes.

A rapid cytoplasmic signaling cascade initiated by activated Ras proteins. As HBx protein strongly elevate the levels of phosphorylated Raf, GTP-bound Ras and tyrosine-phosphorylated and activates MAP kinase [53] that eventually leads to HCC formation in hepatocytes. In another study, it is found that HBx can increase calcium level in mitochondria and promote store-operated Ca^2+^ entry (SOCE) to maintain higher cytosolic calcium level that stimulate HBV replication [54]. HBx can prompt cell cycle regulatory pathways and apoptosis [55]. HBx is found to be linked with many other components of the basic transcriptional machinery, including transcription factor II B (TFIIB), Transcription factor II H (TFIIH), the TATA-binding protein (TBP) and the RPB5 subunit of RNA polymerases [56–58]. HBx strongly mediates apoptosis in certain types of cells. HBx has the ability to inhibit the pRb tumor suppressor function and increase E2F transcription factor1 activity that is a positive cell cycle regulator [59]. Moreover, HBX protein can obstruct cell death mediated by p53, TNF, Fas and TGF-*β* [60, 61]. The HBx can activates the Jak-STAT signaling pathway by acting as an inducer [62] it also breakthrough the liver cancer by down-regulating the dual-specificity protein phosphatase (PTEN) and Phosphatidylinositol 3,4,5-trisphosphate 3-phosphatase, known as a tumor suppressor genes [63]. The HBx protein can provoke the activities of the PI-3K-AKT/PKB, ERK. It was found that matrix metallopeptidase 9 (MMP-9) expression also enhanced by dual transcriptional upregulations of AP-1 and NF-*κ*B [64]. Studies revealed that ribosomal protein S3a (RPS3a) interacts with HBx, and contributes to viral induced oncogenesis by enhancing HBx-induced NF-*κ*B signaling pathway that results in HCC development [65]. *HBx* has been variably reported to activate *STAT3*, *WNT*/*CTNNB1* or bind to and inactivate the TP53 protein [66, 67]. HBx inhibits p53-mediated transcriptional activation and contributes to the molecular pathogenesis of human HCC [68]. Other protein targets for HBx are damaged DNA binding proteins, p53, proteasome subunits; these proteins interacts with the cyclic AMP-responsive element, ATF-2 and basal transcription factors [69]. It has been found that during HBx-induced-HCC many cellular cytoskeletal genes such as microtubule genes tubulinb 2, tubulinb 3, tubulinb 6, keratin 8 (K-8) and keratin 18 (K-18), acting1 (Actg1) and intermediate filament genes periplakin were dysregulated, As it has been documented that these genes were closely clustered and up regulated in liver tissues [70]. The metastasis-associated protein 1 (MTA1) gene is one of the important transcriptional target of HBx protein; It has been found that MTA1 activates hypoxia-inducible factor 1*α* and vascular endothelial growth factor which contributes to angiogenesis in hepatic cancer [71]. The MTA1 gene expression found to be significantly higher in HCC [72]. The HBx protein may increase telomerase reverse transcriptase (TERT) expression as well as telomerase activity. The p21-activated kinase (PAK1) also increase the life-span of hepatocytes and contributing to malignant transformation as well as promote tumor growth [73]. Thus; several transcriptional regulatory genes are activated by the interaction with HBx protein that can eventually lead to HCC (Figure 1).

## 7. Monogenic and Polygenic Risk Factors That Induced HCC

**Figure 2:**
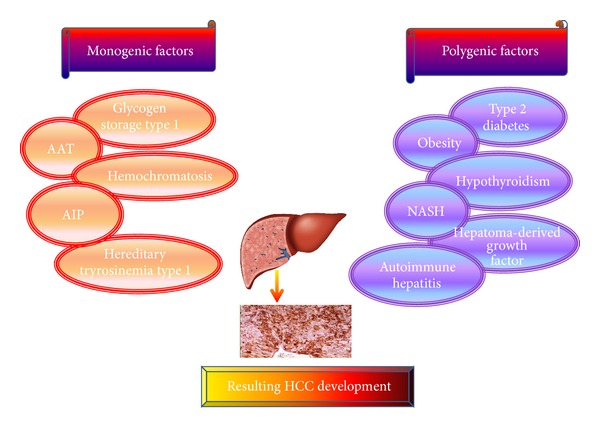
Monogenic and Polygenic diseases causing HCC development.

Primary liver cancer is frequently cause by HBV induced HCC. This form of cancer is different from other forms of hepatic carcinomas. Vaccination is used to prevent HBV induced HCC, but vaccination does not protect those already infected with HBV. Most common risk factors for developing HCC are; alcoholic liver disease, and nonalcoholic steatohepatitis (NASH) [74] intake of aflatoxin contaminated food, diabetes, obesity, certain hereditary conditions such as hemochromatosis, and some metabolic disorders [75–77]. It was found that rare monogenic syndromes, alpha1-antitrypsin (AAT) deficiency and glycogen storage disease type 1 is caused by several mutations in a gene named SERPINA1. This gene was found to encode a serine protease inhibitor. Studies also revealed that over expression of SERPINA1 in liver causes the inhibition of neutrophils elastase. Over expression of SERPINA1 in liver causes the inhibition of neutrophils elastase. Moreover, SERPINA1 also caused glycogen storage disease type 1. Hemochromatosis gene (HFE) is inherited as an autosomal recessive trait. This gene is the cause of iron over load in liver. Both glycogen and iron over load increase the chance of HCC progression. Other known monogenic factors that involve in HCC development are acute intermittent hepatic porphyria (AIP), fumarylaceto acetate hydrolase (FAH) as well as hereditary tyrosinemia type I and Familial porphyria cutanea tarda (PCT). It was found that children with tyrosinemia are at high risk of liver transplantation as beyond age of two years the incidence of HCC increases substantially [78]. A couple of familiar conditions or diseases inherited as polygenic traits, for example, autoimmune hepatitis (AIH), hypothyroidism and type 2 diabetes may also contribute to HCC development [79]. The genetic heterogeneity that is cause by a number of unlinked single gene defects may increase the susceptibility to HCC [80]. It has been found that among men with diabetes, the risk of chronic nonalcoholic liver disease and HCC is doubled [81]. Diabetes mellitus is an independent factor linked with HBV induced HCC [82]. Other factors that were found are obesity, the nonalcoholic fatty liver Disease (NAFLD). Obesity can lead to insulin resistance (IR) and steatosis, both these factors are closely linked with excretion of inflammatory cytokines. Therefore, diabetes and obesity can cause hepatic inflammation and oxidative stress resulting in hepatocyte's injury, subsequently HCC. IL6 and TNF production also up regulated during obesity induced HCC. Thus linking liver steatosis, with inflammation and expression of oncogenic transcription factor STAT3, a phenotype was shared by both virus and nonvirus-related HCCs [83]. It was revealed that Hepatoma-derived growth factor (HDGF) expressed more strongly in the cytoplasm and nucleus of HCC cells as compared to other normal hepatocytes. High expression of HDGF is one of the possible causes of HCC progression [84]. Hereditary hemochromatosis (HH) is concord with an increased risk for HCC. The risk for HCC also increased among the patients of homozygous *β* thalassemia and African iron overload disease [85]. All these monogenic and polygenic factors result in HCC development as depicted in Figure 2.

## 8. Pathways Activated by HBV Infection

**Figure 3:**
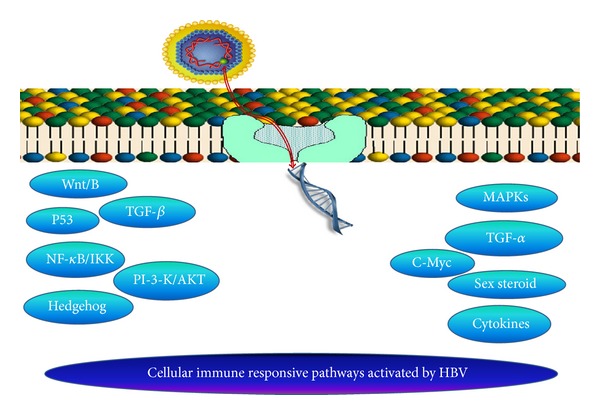
Pathways involve in HBV induced HCC.

Many pathways of cellular immune system are activated during HBV infection. The deregulation of signaling pathways including MAPKs, p53, Sex steroid, Wnt/*β*-catenin, transforming growth factor *β* (TGF*β*), PI3K/AKT, cytokines, IKK/NF-*κ*B and Hedgehog (Hh) were found to be closely related with HCC development. These signaling cascades mostly leads to down-regulation of tumor-suppressor gene and up regulation of tumor-causing genes [120]. It has been studied that both Cytokine lymphotoxin (LT) *α* and *β* and their receptor (LT*β*R) are up regulated in HBV-induced HCC. Sustained LT signaling is another channel involved in HBV-induced HCC [121]. Many signal transduction process that were important for stem cell differentiations and proliferation also deregulated during hepatocarcinogenesis (Table 2) [122]. In hepatocellular carcinoma chromo-domain helicase/ATPase DNA binding protein 1-like gene (CHD1L) was frequently amplified. The CHD1L involves in metastasis by increasing cell motility, tumor cell migration, epithelial-mesenchymal transition (EMT) via ARHGEF9-mediated Cdc42 activation, invasion and inducing filopodia formation. The results obtained indicates that CHD1L-ARHGEF9-Cdc42-EMT might be a novel pathway involved in metastasis and HCC progression [123]. The level of IL6 was found to be increased in HCC cells which proved that IL6 and Inflammatory cytokines, play a significant role is HCC development [51]. Level of IL6 may also predict the shift from viral hepatitis to HCC in humans [124] due to Hh signal activation. It has been documented that the expression of HBx and Hh is highly correlated in human liver cancer cell lines [125]. It was studied that in patients with HBV induced HCC, transforming growth factorbeta (TGF-*β*) cytokine and its isoforms initiates a signaling cascade, which is closely linked to liver cirrhosis and fibrosis. A scientist found that when HBx is over expressed in hepatic cell line then tuberous sclerosis complex 1 (TSC1), I*κ*B kinase *β* (IKK*β*) and mammalian target of rapamycin (mTOR) downstream effector S6 kinase (S6K1) signaling pathways upregulated. Thus, through the use of IKK*β*, HBx deregulates TSC1/mTOR signaling. which is closely linked to HBV-associated HCC development [126]. C-Myc and TGF-*α* were found to induce continued and cumulative transcriptional changes in the liver and starts oncogenesis [127]. One study showed that LSF is a key mediator of the Notch signaling pathway [128]. One group of scientists in China disclosed that transcriptional inactivation of p16 and p15 genes also involves in hepatocarcinogenesis. The p15 and p16 genes inactivation may be caused by 5′ CpG Island methylation in primary HCC [129]. It has been found that three dominant group of genes that were up regulated by HBV heat shock proteins, oxidative and metabolic stress and growth and apoptosis-related genes [130]. The vimentin and IQGAP1 mRNA expression levels increased significantly throughout hepatotumorigenesis provide another target to treat HCC [131]. Targeting the key molecules in the oncogenic signaling pathway might be a promising strategy for HCC therapy (Figure 3).

## 9. Oxidative Stress Induced HCC by HBV

As HBV is a DNA virus which integrates its genome inside the host genome, during HBV infection, viral replication occurs inside infected hepatocytes within viral capsids. In this manner, viral genome conceals itself from pattern recognition receptors (PRRs), of innate immune system, preventing the detection of initial HBV infectious particles [132] PPRs including Toll-like receptors (TLRs) [133, 134] that recognize the pathogen-associated molecular patterns leading to an alter macrophage phenotype. These macrophages secrete reactive oxygen species (ROS); such as type I IFNs (IFN-*α* and IFN-*β*), nitric oxide and other cytokines and chemokines, which may results in up-regulation of intracellular response elements like IRFs, iNOS, NO. it was studied that the general production of nitric oxide and ROS by activated macrophages may also cause hepatocytes destruction [135]. ROS can cause oxidative protein, DNA damage. Oxidative damage to tumor suppressor genes [136]. ROS also effects the central cellular processes such as apoptosis and proliferation leading to the development of cancer [137]. Chronic HBV infection results in increase intracellular iron level in liver. The presence of HBV surface antigen and iron deposition strongly linked with each other [138]. In chronic HBV infected patients, TNF-*α* also boost up causing inflammation in hepatocytes [139]. It has been stated that viral infection induces oxidative stress affect the cellular protein kinase/phosphatase balance, which has been studied in a number of tumors. The reactive oxygen species has direct effects on major cellular processes through the activation of transcription factors, including NF-*κ*B, MAPK and AP-1 pathways [140]. Up regulation of AP-1 results in enhanced cyclin D1 expression and CDKs, known to promote cell division by mitosis. It was narrated that reactive oxygen species activates the NF-*κ*B by cytokines and TNF. Genes that were up-regulated during oxidative stress are aldo-keto reductase 1 family B7 (AKR1B7–) like protein gene, hemoglobin alpha (HBA1) and beta (HBB) [141, 142] Oxidative Stress responsive Genes APOE, ATOX1, and CAT [143–145] were found to be activated during infection. Genes that involve in DNA damage are CCND1, CDKN1A (p21CIP1/WAF1), MSH2, MSH3, TP53, and XIAP [146, 147], along with modified gene expression and mutations are all required participants in the process of carcinogenesis. It was found that oxidative stress is associated with hepatitis B activity and XRCC1 gene is putatively associated with DNA damage [148]. Although all these events derived by different gene products, a common theme which is the entanglement of ROS that cause oxidative stress. It was studied that CD8 T-cells effectively control HBV replication, especially in the presence of IFN-gamma and TNF-alpha [149]. The platelets Causes hepatic injury by promoting accumulation of virus-specific CD8 (+) T cells. Previous data about CD8^+^ shows that although it works to control HBV viral particle clearance and replication, but it may also cause liver injury by recruiting nonviral specific T-cells [150]. As studies revealed that immune mediated necro-inflammation is another cause of HCC development [151]. It was found that CD8^+^ T cell causes hepatic inflammation derive by INF gamma [152]. It was demonstrated that metabolic syndrome (MS) was associated with both cryptogenic cirrhosis and nonalcoholic steatohepatitis, Currently MS can be consider as an independent and major risk factor for CHB infection related Cirrhosis [153–155] and HCC development [156]. The risk of HCC was found positively correlated with the number of MS components found in patients not chronically infected with HBV [157].

## 10. Role of cccDNA in HBV-Related HCC Recurrence

It has been exposed by a team of scientist that HBV cccDNA is a unique intermediate that serve as a template for the production of HBV pregenomic RNA (pgRNA) that in return responsible for the persistent HBV infection in hepatocytes. It was found that any piece of pgRNA can be act as a template for minus DNA strand synthesis [158–160]. The cccDNA level increases unexpectedly in the initial phase of proliferation after that the level of cccDNA decreased dramatically in the cells during cell division due to loss of extra-chromosomal plasmid DNA [161] thus cccDNA level shows dynamic expression in different phases of cell growth. The pegylated interferon alpha-2b (peg-IFN) and adefovir dipivoxil (ADV) antiviral therapy led to considerably decreased the cccDNA levels by a primarily noncytolytic mechanism [158, 162]. The peginterferon alpha-2b, lamivudine treatment also results in decrease level of HBsAg that is correlated with cccDNA level [163]. Some patients also treated with tenofovir disoproxil fumarate (TDF) which shows incomplete or low responce to ADV therapy [164]. A positive correlation was found between cccDNA and HBcrAg at the incidence of HCC reoccurance, thus cccDNA is a positive haalmark of HBV related HCC reoccurance [165]. It was reported in ScienceDaily that IFN*α* can cause silencing of cccDNA as well as Lymphtoxin beta receptor (LTbR) agonisation and also can provide novel alternative therapeutic approach for curing CHB infection [166].

## 11. Promising Therapies for HBV Induced HCC

### 11.1. Diagnosis

HCC due to HBV is a lethal disease as the diagnosis is late in this type of cancer, best method, untill now for the diagnosis of HCC is computed tomography (CT) of the liver, Magnetic resonance imaging (MRI) with contrast enhancement is most common method for detecting liver inflammation. Liver angiography with lipiodol injection and follow by CT is another accurate method for detecting liver lesions. Liver biopsy is seldom required for diagnosis.

## 12. Antiviral and Nonsurgical Treatments for HCC

Effective antiviral therapies are now available. IFN-*α* therapy was proven to be efficient in reducing the risk of HCC recurrence patients with small HCC [167]. Continued suppression of HBV replication with anti-nucleoside or anti-nucleotide or by analogs may decrease the risk of HBV-related HCC development; it was found that Long-term lamivudine treatment can prevent complications of HBV-related liver disease. However, when lamivudine does not affect than entecavir and Adefovir dipivoxil have been shown to be more effective and safe for the treatment of patients with Chronic hepatitis [168]. Patients who developed adefovir resistance will respond only to adefovir monotherapy rather than lamivudine and adefovir combination therapy [169]. Angiogenesis inhibition is a natural therapeutic target for all solid tumors, targeted agent sorafenib provided proof-of-concept for molecularly targeted therapy in advanced HCC, tyrosine kinase inhibitor (TKIs) such as brivanib, everolimus, and monoclonal antibodies (e.g., ramucirumab) erlotinib, sunitinib, vandetanib, cediranib, brivanib, foretinib, and dovitinib [170] are being tested as second-line therapies [171], brivanib is a dual fibroblast growth factor pathway and VEGF receptor inhibitor, additional agent for the treatment of patients with HCC includes, ramucirumab, ABT-869, bevacizumab, ARQ-197 and everolimus [172]. The early evidence of anti-tumor activity was shown by sunitinib. Despite significant progress, progressive HCC remains an incurable disease; combination of molecular targeted approach will be helpful in management of advanced disease. Transarterial chemoembolization has been tested and found significantly important in increasing survival, in highly selected patients with good liver function [173]. Hormonal therapy and biotherapy, radiotherapy, thermal and laser ablation, percutaneous alcohol injection, cryoablation, as well as radiofrequency ablation (RFA) percutaneous ethanol injection (PEI) and systemic therapy are also potentially effective curative [174, 175].

## 13. Recent Advancement in HBV Research

Recently, in a new study by Alasdair Steven (Chief of the NIAMS Laboratory of Structural Biology Research) and Paul Wingfield (Chief of the NIAMS Protein Expression Laboratory) a unique antibody that stably binds e-antigen was developed. Understanding the structure of e-antigen will be helpful in future studies. As this antigen plays a significant role in HBV persistence. It was elucidated that full surface vaccines gives stronger immunity and are good even for neonates [176].

## 14. Conclusion

In conclusion, the present review indicates that HBV infection is in one of the major cause of HCC development. HBV protein S, C, P and X are responsible for viral replication and activation of several cell signaling pathways such as NF-*κ*B, PI-3K and Hedgehog and eventually lead to HCC. Moreover, a couple of polygenic and monogenic pathways such as NASH, Obesity, and Type 2 Diabetes and Glycogen Storage disease type 1, Hemochromatosis respectively were also involved in HCC development. Targeting the key element involves in different signaling pathways that are activated by HBV infection and genetic factors will be helpful in future to find the easy and permanent cure from HCC. Moreover, the information regarding up-regulation and downregulation of genes during HCC will be helpful to find noninvasive markers in blood.

## Figures and Tables

**Table 1:** Genes upregulated during HBV-induced Hepatocellular Carcinoma

Genes up-regulated during HCC				
Gene name	Gene	Function	Activation by	Reference
B-cell lymphoma/leukemia 2	BCL2	Apoptosis-related genes	HBV pre-S2 increased Bcl-2 expression	[86]
Baculoviral IAP repeat containing 5 (survivin)	BIRC5	Cell cycle, regulation, apoptosis inhibitor	HBV and HCV	[87]
Cyclin D1	CCND1	Regulators of CDK kinases, interact with tumor suppressor gene	HBV	[88]
CASP8 and FADD-like apoptosis regulator	CFLAR	Apoptosis-related genes	HBV-induced HCC	[89]
Type II keratin Kb8	*KRT8 *	Cytoskeletal organization, cirrhosis	HBV	[89]
Ribosomal protein S5	*RPS5 *	Protein synthesis	HBV	[89]
Insulin-like growth factor binding protein 2	*IGFBP2 *	Cell membrane receptor related genes	HBV	[89]
Matrix metalloproteinase 9	*MMP9 *	Metastasis-related genes	HBV	[89]
ATP synthase F 1	*ATP5F1 *	Transportion	HBV	[89]
Frizzled-7 receptor	FZD7	Activates the Wnt/beta-catenin pathway	HBV	[90]
Insulin-like growth factor2	IGF2	Stimulatory role, gestation	HBV	[91]
Maternally-transmitted human gene	*H19 *	Cancer causing	HBV	[91]
Tumor growth factor beta 1	TGFB1	Tumor suppressor	HBV	[92]
Induced myeloid leukemia cell differentiation protein	Mcl-1	Controlling life and death decisions in response to rapidly changing environmental cues and immune response	*HBV* pre-S2Δ protein	[86]
Transforming growth factor alpha	TGFA	Morphogenesis	HBV	[93]
Lymphoid enhancer-binding factor 1	LEF1	Regulatory proteins and potential drug target	Chronic HBV	[94]
Nuclear factor kappa B	NFKB1	Inflammation, immunity, differentiation, tumorigenesis, and apoptosis	HBV	[95]
Insulin receptor substrate 1	IRS1		HBV	[96]
Peptidyl-prolyl cis-trans isomerase NIMA-interacting 1	PIN1	Cell proliferation, cell survival, immune responses	HBV	[97]
Hepatocyte growth factor receptor (HGFR)	MET	Protooncogene, angiogenesis, gastrulation,	HBV	[98]
Protein tyrosine kinase 2	PTK2	regulation of the tumor suppressor p53	HBV and HCV	[99]
Ras-related C3 botulinum toxin substrate 1	RAC1	Cell growth, cytoskeletal, activation of protein kinases	HBV	[91]
Ras homolog gene family, member A	RHOA	Regulation and timing of cell division.	HBV	[91]
Mothers against decapentaplegic homolog 7	SMAD7	Overexpression causes cancer	HBV	[100]
Immunoglobulin transcription factor 2	TCF4	Immune responce	HBV	[101]
TNF-related apoptosis-inducing ligand (TRAIL)	TNFSF10	Activates NFkappaB	HBV	[102]
X-linked inhibitor of apoptosis protein	XIAP	Inhibits caspase 3, 7, and 9	HBV	[103]
Proto-oncogene tyrosine-protein kinase	SRC	Regulation of embryonic development and cell growth.	HBV	[47]
Store-operated Ca(2+) entry	SOCE	Preventing the overload of the cell with excessive Ca(2+) ions	HBx	[104]
Metastasis associated 1	MTA	Contributes to angiogenesis	HBV	[68]
BAX	Upregulated by HBx	Apoptotic activity	Bcl-2–associated X protein	[105]

**Table 2:** Genes down regulated during HBV induced hepatocellular carcinoma

Genes Down regulated during HCC:				
Gene	Gene name	Activated by	Function	Reference
BID	BH3 interacting domain	HBx protein	Cell death regulation	[106]
P53	Protein 53	HBx	Tumor suppressor	[107]
p21WAF1	Protein 21	HBx	Stress response, acts with p53	[107]
IGFBP3	Insulin-like growth factor-binding protein 3	HBx		[107]
CASP8	Caspase 8	HBV	Apoptosis-related cysteine peptidase	[89]
CDKN1A/p21	Cyclin-dependent kinase inhibitor 1A	HBV	Inhibits the activity of cyclin-CDK2	[107]
DLC1	Deleted in liver cancer 1	HBV	Tumor suppressor, cell growth, and proliferation	[108]
FADD	Fas-associated protein with death domain	HBV	Adaptor molecule that bridges the Fas-receptor	[109]
IGFBP1	Insulin-like growth factor-binding protein 1	HBV	Tumor suppressor	[110]
ITGB1	Integrins beta 1	HBV	Embryogenesis, hemostasis, tissue repair, immune response	[111]
Hhip	Hh-interacting protein	HBV	Modulates hedgehog signaling	[112]
PTEN		HBV	Apoptosis, cell movement	[113]
RB1	Retinoblastoma 1	HBV	Oncogenic, tumor suppressor	[114]
SMAD4		HBV	Transmitting chemical signal, regulates cell growth and division	[115]
SOCS1	Suppressor of cytokine signaling 1	HBV	Negative regulator in TNF-induced inflammation and activation of c-jun N-terminal kinase	[116]
SOCS3	Suppressor of cytokine signaling 3	HBV		[117]
TGFBR2	Transforming growth factor, beta receptor II	HBV	Transmits signals, stimulation of cell growth and division, differentiation	[118]
CDKN2A	Cyclin-dependent kinase inhibitor 2A	HBV and HCV	Cell cycle control, tumor suppressor gene	[119]

## References

[1] Lin C-L, Kao J-H (2011). The clinical implications of hepatitis B virus genotype: recent advances. *Journal of Gastroenterology and Hepatology*.

[2] Summers J (1981). Three recently described animal virus models for human hepatitis B virus. *Hepatology*.

[3] Stuyver L, de Gendt S, van Geyt C (2000). A new genotype of hepatitis B virus: complete genome and phylogenetic relatedness. *Journal of General Virology*.

[4] Arauz-Ruiz P, Norder H, Robertson BH, Magnius LO (2002). Genotype H: a new Amerindian genotype of hepatitis B virus revealed in Central America. *Journal of General Virology*.

[5] Viana R, Wang R, Yu MC, Welschinger R, Chen C-Y, Kew MC (2009). Hepatitis B viral loads in southern African blacks with hepatocellular carcinoma. *Journal of Medical Virology*.

[6] Kew MC (2010). Hepatocellular carcinoma in African Blacks: recent progress in etiology and pathogenesis. *World Journal of Hepatology*.

[7] Mason CSaWS (2000). Hepatitis B virus biology. *Microbiology and Molecular Biology Reviews*.

[8] Wu S, Kanda T, Imazeki F (2010). Hepatitis B virus e antigen downregulates cytokine production in human hepatoma cell lines. *Viral Immunology*.

[9] Di Bisceglie AM (2009). Hepatitis B and hepatocellular carcinoma. *Hepatology*.

[10] Liu K, Lei X-Z, Zhao L-S (2005). Tissue microarray for high-throughput analysis of gene expression profiles in hepatocellular carcinoma. *World Journal of Gastroenterology*.

[11] Milich D, Liang TJ (2003). Exploring the biological basis of hepatitis B e antigen in hepatitis B virus infection. *Hepatology*.

[12] Zhang Z, Torii N, Hu Z, Jacob J, Liang TJ (2001). X-deficient woodchuck hepatitis virus mutants behave like attenuated viruses and induce protective immunity *in vivo*. *Journal of Clinical Investigation*.

[13] Hu Z, Zhang Z, Kim JW, Huang Y, Liang TJ (2006). Altered proteolysis and global gene expression in hepatitis B virus X transgenic mouse liver. *Journal of Virology*.

[14] Bouchard MJ, Schneider RJ (2004). The enigmatic X gene of hepatitis B virus. *Journal of Virology*.

[15] Jiang Z, Jhunjhunwala S, Liu J (2012). The effects of hepatitis B virus integration into the genomes of hepatocellular carcinoma patients. *Genome Research*.

[16] Shafritz DA, Shouval D, Sherman HI, Hadziyannis SJ, Kew MC (1981). Integration of hepatitis B virus DNA into the genome of liver cells in chronic liver disease and hepatocellular carcinoma. Studies in percutaneous liver biopsies and post-mortem tissue specimens. *New England Journal of Medicine*.

[17] Tu H, Bonura C, Giannini C (2001). Biological impact of natural COOH-terminal deletions of hepatitis B virus X protein in hepatocellular carcinoma tissues. *Cancer Research*.

[18] Kremsdorf D, Soussan P, Paterlini-Brechot P, Brechot C (2006). Hepatitis B virus-related hepatocellular carcinoma: paradigms for viral-related human carcinogenesis. *Oncogene*.

[19] Murakami Y, Saigo K, Takashima H (2005). Large scaled analysis of hepatitis B virus (HBV) DNA integration in HBV related hepatocellular carcinomas. *Gut*.

[20] Su I-J, Wang H-C, Wu H-C, Huang W-Y (2008). Ground glass hepatocytes contain pre-S mutants and represent preneoplastic lesions in chronic hepatitis B virus infection. *Journal of Gastroenterology and Hepatology*.

[21] Soussan P, Garreau F, Zylberberg H, Ferray C, Brechot C, Kremsdorf D (2000). *In vivo* expression of a new hepatitis B virus protein encoded by a spliced RNA. *Journal of Clinical Investigation*.

[22] Fang Z-L, Sabin CA, Dong B-Q (2008). Hepatitis B virus pre-S deletion mutations are a risk factor for hepatocellular carcinoma: a matched nested case—control study. *Journal of General Virology*.

[23] Huang H-P, Hsu H-Y, Chen C-L (2010). Pre-S2 deletions of hepatitis B virus and hepatocellular carcinoma in children. *Pediatric Research*.

[24] Hsieh Y-H, Su I-J, Wang H-C (2004). Pre-S mutant surface antigens in chronic hepatitis B virus infection induce oxidative stress and DNA damage. *Carcinogenesis*.

[25] Hsieh YH, Su IJ, Yen CJ (2012). Histone deacetylase inhibitor suberoylanilide hydroxamic acid suppresses the pro-oncogenic effects induced by hepatitis B virus pre-S2 mutant oncoprotein and represents a potential chemopreventive agent in high-risk chronic HBV patients. *Carcinogenesis*.

[26] Bréchot C (2004). Pathogenesis of hepatitis B virus-related hepatocellular carcinoma: old and new paradigms. *Gastroenterology*.

[27] Chen WN, Chen JY, Jiao BY (2012). Interaction of the hepatitis B spliced protein with cathepsin B promotes hepatoma cell migration and invasion. *Journal of Virology*.

[28] Hwang G-Y, Lin C-Y, Huang L-M (2003). Detection of the hepatitis B virus X protein (HBx) antigen and anti-HBx antibodies in cases of human hepatocellular carcinoma. *Journal of Clinical Microbiology*.

[29] Gearhart TL, Bouchard MJ (2010). The hepatitis B virus X protein modulates hepatocyte proliferation pathways to stimulate viral replication. *Journal of Virology*.

[30] Dandri M, Schirmacher P, Rogler CE (1996). Woodchuck hepatitis virus X protein is present in chronically infected woodchuck liver and woodchuck hepatocellular carcinomas which are permissive for viral replication. *Journal of Virology*.

[31] Doria M, Klein N, Lucito R, Schneider RJ (1995). The hepatitis B virus HBx protein is a dual specificity cytoplasmic activator of Ras and nuclear activator of transcription factors. *The EMBO Journal*.

[32] Sirma H, Weil R, Rosmorduc O (1998). Cytosol is the prime compartment of hepatitis B virus X protein where it colocalizes with the proteasome. *Oncogene*.

[33] Weil R, Sirma H, Giannini C (1999). Direct association and nuclear import of the hepatitis B virus X protein with the NF-*κ*B inhibitor I*κ*B*α*. *Molecular and Cellular Biology*.

[34] Forgues M, Marrogi AJ, Spillare EA (2001). Interaction of the hepatitis B virus X protein with the Crm1-dependent nuclear export pathway. *Journal of Biological Chemistry*.

[35] Zhang X, Zhang H, Ye L (2006). Effects of hepatitis B virus X protein on the development of liver cancer. *Journal of Laboratory and Clinical Medicine*.

[36] Galibert F, Mandart E, Fitoussi F, Tiollais P, Charnay P (1979). Nucleotide sequence of the hepatitis B virus genome (subtype ayw) cloned in *E. coli*. *Nature*.

[37] Miller RH, Robinson WS (1986). Common evolutionary origin of hepatitis B virus and retroviruses. *Proceedings of the National Academy of Sciences of the United States of America*.

[38] Koike K, Moriya K, Yotsuyanagi H, Iino S, Kurokawa K (1994). Induction of cell cycle progression by hepatitis B virus HBx gene expression in quiescent mouse fibroblasts. *Journal of Clinical Investigation*.

[39] Nakatake H, Chisaka O, Yamamoto S, Matsubara K, Koshy R (1993). Effect of X protein on transactivation of hepatitis B virus promoters and on viral replication. *Virology*.

[40] Spandau DF, Lee C-H (1988). Trans-activation of viral enhancers by the hepatitis B virus X protein. *Journal of Virology*.

[41] Twu J-S, Chu K, Robinson WS (1989). Hepatitis B virus X gene activates *κ*B-like enhancer sequences in the long terminal repeat of human immunodeficiency virus 1. *Proceedings of the National Academy of Sciences of the United States of America*.

[42] Zahm P, Hofschneider PH, Koshy R (1988). The HBV X-ORF encodes a transactivator: a potential factor in viral hepatocarcinogenesis. *Oncogene*.

[43] Chen H-S, Kaneko S, Girones R (1993). The woodchuck hepatitis virus X gene is important for establishment of virus infection in woodchucks. *Journal of Virology*.

[44] Kekule AS, Lauer U, Weiss L, Luber B, Hofschneider PH (1993). Hepatitis B virus transactivator HBx uses a tumour promoter signalling pathway. *Nature*.

[45] Kim C-M, Koike K, Saito I, Miyamura T, Jay G (1991). HBx gene of hepatitis B virus induces liver cancer in transgenic mice. *Nature*.

[46] Koike K, Moriya K, Iino S (1994). High-level expression of hepatitis B virus HBx gene and hepatocarcinogenesis in transgenic mice. *Hepatology*.

[47] Moriarty AM, Alexander H, Lerner RA, Thornton GB (1985). Antibodies to peptides detect new hepatitis B antigen: serological correlation with hepatocellular carcinoma. *Science*.

[48] Rossner MT (1992). Review: hepatitis B virus X-gene product: a promiscuous transcriptional activator. *Journal of Medical Virology*.

[49] Seto E, Mitchell PJ, Benedict Yen TS (1990). Transactivation by the hepatitis B virus X protein depends on AP-2 and other transcription factors. *Nature*.

[50] Shirakata Y, Kawada M, Fujiki Y (1989). The X gene of hepatitis B virus induced growth stimulation and tumorigenic transformation of mouse NIH3T3 cells. *Japanese Journal of Cancer Research*.

[51] Li N, Grivennikov SI, Karin M (2011). The unholy trinity: inflammation, cytokines, and STAT3 shape the cancer microenvironment. *Cancer Cell*.

[52] Gearhart TL, Bouchard MJ (2010). The hepatitis B virus X protein modulates hepatocyte proliferation pathways to stimulate viral replication. *Journal of Virology*.

[53] Yang B, Bouchard MJ (2012). The hepatitis B virus X protein elevates cytosolic calcium signals by modulating mitochondrial calcium uptake. *Journal of Virology*.

[54] Bouchard MJ, Schneider RJ (2004). The enigmatic X gene of hepatitis B virus. *Journal of Virology*.

[55] Cheong J-H, Yi M-K, Lin Y, Murakami S (1995). Human RPB5, a subunit shared by eukaryotic nuclear RNA polymerases, binds human hepatitis B virus X protein and may play a role in X transactivation. *The EMBO Journal*.

[56] Haviv I, Shamay M, Doitsh G, Shaul Y (1998). Hepatitis B virus pX targets TFIIB in transcription coactivation. *Molecular and Cellular Biology*.

[57] Lin Y, Nomura T, Cheong J, Dorjsuren D, Iida K, Murakami S (1997). Hepatitis B virus X protein is a transcriptional modulator that communicates with transcription factor IIB and the RNA polymerase II subunit 5. *Journal of Biological Chemistry*.

[58] Murakami S, Cheong J, Ohno S, Matsushima K, Kaneko S (1994). Transactivation of human hepatitis B virus X protein, HBx, operates through a mechanism distinct from protein kinase C and okadaic acid activation pathways. *Virology*.

[59] Byung Hyune Choi BHC, Choi M, Hyun Yong Jeon HYJ, Hyune Mo Rho HMR (2001). Hepatitis B viral X protein overcomes inhibition of E2F1 activity by pRb on the human Rb gene promoter. *DNA and Cell Biology*.

[60] Qadri I, Conaway JW, Conaway RC, Schaack J, Siddiqui A (1996). Hepatitis B virus transactivator protein, HBx, associates with the components of TFIIH and stimulates the DNA helicase activity of TFIIH. *Proceedings of the National Academy of Sciences of the United States of America*.

[61] Elmore LW, Hancock AR, Chang S-F (1997). Hepatitis B virus X protein and p53 tumor suppressor interactions in the modulation of apoptosis. *Proceedings of the National Academy of Sciences of the United States of America*.

[62] Pan J, Duan L-X, Sun BS, Feitelson MA (2001). Hepatitis B virus X protein protects against anti-Fas-mediated apoptosis in human liver cells by inducing NF-*κ*B. *Journal of General Virology*.

[63] Nicolas M, Wolfer A, Raj K (2003). Notch1 functions as a tumor suppressor in mouse skin. *Nature Genetics*.

[64] Yeh C-T (2000). Hepatitis B virus X protein: searching for a role in hepatocarcinogenesis. *Journal of Gastroenterology and Hepatology*.

[65] Lim K-H, Kim K-H, Choi SI (2011). RPS3a over-expressed in HBV-associated hepatocellular carcinoma enhances the HBx-induced NF-*κ*B signaling via its novel chaperoning function. *PLoS ONE*.

[66] Shih W-L, Kuo M-L, Chuang S-E, Cheng A-L, Doong S-L (2000). Hepatitis b virus x protein inhibits transforming growth factor-*β*-induced apoptosis through the activation of phosphatidylinositol 3-kinase pathway. *Journal of Biological Chemistry*.

[67] Lee Y-H, Yun Y (1998). HBx protein of hepatitis B virus activates Jak1-STAT signaling. *Journal of Biological Chemistry*.

[68] Chung T-W, Lee Y-C, Kim C-H (2004). Hepatitis B viral HBx induces matrix metalloproteinase-9 gene expression through activation of ERK and PI-3K/AKT pathways: involvement of invasive potential. *The FASEB Journal*.

[69] Bock C-T, Toan NL, Koeberlein B (2009). Subcellular mislocalization of mutant hepatitis B X proteins contributes to modulation of stat/socs signaling in hepatocellular carcinoma. *Intervirology*.

[70] Sun Q, Wang Y, Zhang Y (2007). Expression profiling reveals dysregulation of cellular cytoskeletal genes in HBx-induced hepatocarcinogenesis. *Cancer Biology & Therapy*.

[71] Lee S-W, Lee YM, Bae S-K, Murakami S, Yun Y, Kim K-W (2000). Human hepatitis B virus x protein is a possible mediator of hypoxia-induced angiogenesis in hepatocarcinogenesis. *Biochemical and Biophysical Research Communications*.

[72] Yoo Y-G, Na T-Y, Seo H-W (2008). Hepatitis B virus X protein induces the expression of MTA1 and HDAC1, which enhances hypoxia signaling in hepatocellular carcinoma cells. *Oncogene*.

[73] Xu J, Liu H, Chen L (2012). Hepatitis B virus X protein confers resistance of hepatoma cells to anoikis by up-regulating and activating p21-activated kinase 1. *Gastroenterology*.

[74] El-Serag HB, Rudolph KL (2007). Hepatocellular carcinoma: epidemiology and molecular carcinogenesis. *Gastroenterology*.

[75] Poon D, Anderson BO, Chen L-T (2009). Management of hepatocellular carcinoma in Asia: consensus statement from the Asian Oncology Summit 2009. *The Lancet Oncology*.

[76] Montalto G, Cervello M, Giannitrapani L, Dantona F, Terranova A, Castagnetta LAM (2002). Epidemiology, risk factors, and natural history of hepatocellular carcinoma. *Annals of the New York Academy of Sciences*.

[77] Gomaa AI, Khan SA, Toledano MB, Waked I, Taylor-Robinson SD (2008). Hepatocellular carcinoma: epidemiology, risk factors and pathogenesis. *World Journal of Gastroenterology*.

[78] Weinberg AG, Mize CE, Worthen HG (1976). The occurence of hepatoma in the chronic form of hereditary tyrosinemia. *Journal of Pediatrics*.

[79] Hashimoto E, Yatsuji S, Tobari M (2009). Hepatocellular carcinoma in patients with nonalcoholic steatohepatitis. *Journal of Gastroenterology*.

[80] Dragani TA (2010). Risk of HCC: genetic heterogeneity and complex genetics. *Journal of Hepatology*.

[81] El-Serag HB, Tran T, Everhart JE (2004). Diabetes Increases the Risk of Chronic Liver Disease and Hepatocellular Carcinoma. *Gastroenterology*.

[82] Gao C, Zhao H-C, Li J-T, Yao S-K (2010). Diabetes mellitus and hepatocellular carcinoma: comparison of Chinese patients with and without HBV-related cirrhosis. *World Journal of Gastroenterology*.

[83] Park EJ, Lee JH, Yu G-Y (2010). Dietary and genetic obesity promote liver inflammation and tumorigenesis by enhancing IL-6 and TNF expression. *Cell*.

[84] Yoshida K, Nakamura H, Okuda Y (2003). Expression of hepatoma-derived growth factor in hepatocarcinogenesis. *Journal of Gastroenterology and Hepatology*.

[85] Kowdley KV (2004). Iron, hemochromatosis, and hepatocellular carcinoma. *Gastroenterology*.

[86] Hung J-H, Teng Y-N, WangLily LH-C (2011). Induction of Bcl-2 expression by hepatitis B virus pre-S2 mutant large surface protein resistance to 5-fluorouracil treatment in Huh-7 cells. *PLoS ONE*.

[87] Wang L, Huang J, Jiang M, Sun L (2011). Survivin (BIRC5) cell cycle computational network in human no-tumor hepatitis/cirrhosis and hepatocellular carcinoma transformation. *Journal of Cellular Biochemistry*.

[88] Patil MA, Lee SA, Macias E (2009). Role of cyclin D1 as a mediator of c-Met- and B-catenin-induced hepatocarcinogenesis. *Cancer Research*.

[89] Lee C-F, Ling Z-Q, Zhao T, Lee K-R (2008). Distinct expression patterns in hepatitis B virus- and hepatitis C virus-infected hepatocellular carcinoma. *World Journal of Gastroenterology*.

[90] Kim M, Lee HC, Tsedensodnom O (2008). Functional interaction between Wnt3 and Frizzled-7 leads to activation of the Wnt/*β*-catenin signaling pathway in hepatocellular carcinoma cells. *Journal of Hepatology*.

[91] Iizuka N, Oka M, Tamesa T, Hamamoto Y, Yamada-Okabe H (2004). Imbalance in expression levels of insulin-like growth factor 2 and H19 transcripts linked to progression of hepatocellular carcinoma. *Anticancer Research*.

[92] Migita K, Miyazoe S, Maeda Y (2005). Cytokine gene polymorphisms in Japanese patients with hepatitis B virus infection—association between TGF-*β*1 polymorphisms and hepatocellular carcinoma. *Journal of Hepatology*.

[93] Zhang J, Wang W-L, Li Q, Qiao Q (2004). Expression of transforming growth factor-*α* and hepatitis B surface antigen in human hepatocellular carcinoma tissues and its significance. *World Journal of Gastroenterology*.

[94] Tian X, Li J, Ma Z-M, Zhao C, Wan D-F, Wen Y-M (2009). Role of hepatitis B surface antigen in the development of hepatocellular carcinoma: regulation of lymphoid enhancer-binding factor 1. *Journal of Experimental & Clinical Cancer Research*.

[95] He Y, Zhang H, Yin J (2009). IkappaBalpha gene promoter polymorphisms are associated with hepatocarcinogenesis in patients infected with hepatitis B virus genotype C. *Carcinogenesis*.

[96] Tanaka S, Wands JR (1996). Insulin receptor substrate 1 overexpression in human hepatocellular carcinoma cells prevents transforming growth factor *β*1-induced apoptosis. *Cancer Research*.

[97] Segat L, Milanese M, Crovella S (2007). Pin1 promoter polymorphisms in hepatocellular carcinoma patients. *Gastroenterology*.

[98] Maass T, Sfakianakis I, Staib F, Krupp M, Galle PR, Teufel A (2010). Microarray-based gene expression analysis of hepatocellular carcinoma. *Current Genomics*.

[99] Berzsenyi MD, Roberts SK, Beard MR (2006). Genomics of hepatitis B and C infections: diagnostic and therapeutic applications of microarray profiling. *Antiviral Therapy*.

[100] Teufel A, Staib F, Kanzler S, Weinmann A, Schulze-Bergkamen H, Galle PR (2007). Genetics of hepatocellular carcinoma. *World Journal of Gastroenterology*.

[101] Cha M-Y, Kim C-M, Park Y-M, Ryu W-S (2004). Hepatitis B virus X protein is essential for the activation of Wnt/*β*-catenin signaling in hepatoma cells. *Hepatology*.

[102] Janssen HLA, Higuchi H, Abdulkarim A, Gores GJ (2003). Hepatitis B virus enhances tumor necrosis factor-related apoptosis-inducing ligand (TRAIL) cytotoxicity by increasing TRAIL-R1/death receptor 4 expression. *Journal of Hepatology*.

[103] Zhang X, Dong N, Yin L (2005). Hepatitis B virus X protein upregulates survivin expression in hepatoma tissues. *Journal of Medical Virology*.

[104] Lu J-W, Hsia Y, Yang W-Y (2012). Identification of the common regulators for hepatocellular carcinoma induced by hepatitis B virus X antigen in a mouse model. *Carcinogenesis*.

[105] Ali M, Idrees M, Ali L (2011). Hepatitis B virus in Pakistan: a systematic review of prevalence, risk factors, awareness status and genotypes. *Virology Journal*.

[106] Chen GG, Lai PBS, Chan PKS (2001). Decreased expression of Bid in human hepatocellular carcinoma is related to hepatitis B virus X protein. *European Journal of Cancer*.

[107] Wu Z-J, Zhu Y, Huang D-R, Wang Z-Q (2010). Constructing the HBV-human protein interaction network to understand the relationship between HBV and hepatocellular carcinoma. *Journal of Experimental and Clinical Cancer Research*.

[108] Wong C-M, Lee JM-F, Ching Y-P, Jin D-Y, Ng IO-L (2003). Genetic and epigenetic alterations of DLC-1 gene in hepatocellular carcinoma. *Cancer Research*.

[109] Peng Z, Zhang Y, Gu W (2005). Integration of the hepatitis B virus X fragment in hepatocellular carcinoma and its effects on the expression of multiple molecules: a key to the cell cycle and apoptosis. *International Journal of Oncology*.

[110] Gong Y, Cui L, Minuk GY (2000). The expression of insulin-like growth factor binding proteins in human hepatocellular carcinoma. *Molecular and Cellular Biochemistry*.

[111] Li H, Ge C, Zhao F (2011). Hypoxia-inducible factor 1 alpha-activated angiopoietin-like protein 4 contributes to tumor metastasis via vascular cell adhesion molecule-1/integrin *β*1 signaling in human hepatocellular carcinoma. *Hepatology*.

[112] Choi SS, Omenetti A, Syn W-K, Diehl AM (2011). The role of Hedgehog signaling in fibrogenic liver repair. *International Journal of Biochemistry & Cell Biology*.

[113] Yuan K, Lian Z, Sun B, Clayton MM, Ng IOL, Feitelson MA (2012). Role of mir-148a in hepatitis B associated hepatocellular carcinoma. *PLoS ONE*.

[114] Moghaddam SJ, Haghighi EN, Samiee S (2007). Immunohistochemical analysis of p53, cyclinD1, RB1, c-fos and N-ras gene expression in hepatocellular carcinoma in Iran. *World Journal of Gastroenterology*.

[115] Yao L, Li FJ, Tang ZQ, Gao S, Wu QQ (2012). Smad4 expression in hepatocellular carcinoma differs by hepatitis status. *Asian Pacific Journal of Cancer Prevention*.

[116] Chu P-Y, Yeh C-M, Hsu NC, Chang Y-S, Chang J-G, Yeh K-T (2010). Epigenetic alteration of the SOCS1 gene in hepatocellular carcinoma. *Swiss Medical Weekly*.

[117] Nakagawa H, Maeda S (2012). Inflammation- and stress-related signaling pathways in hepatocarcinogenesis. *World Journal of Gastroenterology*.

[118] Mamiya T, Yamazaki K, Masugi Y (2010). Reduced transforming growth factor-*β* receptor II expression in hepatocellular carcinoma correlates with intrahepatic metastasis. *Laboratory Investigation*.

[119] Csepregi A, Ebert MPA, Röcken C (2010). Promoter methylation of CDKN2A and lack of p16 expression characterize patients with hepatocellular carcinoma. *BMC Cancer*.

[120] Hong YW, Ding J (2011). Molecular signaling in hepatocellular carcinoma. *Cancer Genetics*.

[121] Haybaeck J, Zeller N, Wolf MJ (2009). A lymphotoxin-driven pathway to hepatocellular carcinoma. *Cancer Cell*.

[122] Sameh MA, He R (2011). Liver cancer stem cells. *International Journal of Hepatology*.

[123] Teoh NC (2010). Pre-“EMT”ing key processes in liver carcinogenesis: growing evidence for how malignant hepatocytes invade and conquer: commentary. *Hepatology*.

[124] Wong VW-S, Yu J, Cheng AS-L (2009). High serum interleukin-6 level predicts future hepatocellular carcinoma development in patients with chronic hepatitis B. *International Journal of Cancer*.

[125] Arzumanyan A, Sambandam V, Clayton M (2012). Hedgehog signaling blockade delays hepatocarcinogenesis induced by Hepatitis B virus X protein (HBx). *Cancer Research*.

[126] Yen C-J, Lin Y-J, Yen C-S (2012). Hepatitis B virus X protein upregulates mTOR signalingthrough IKK*β* to increase cell proliferation and VEGF production in hepatocellular carcinoma. *PLoS One*.

[127] Coulouarn C, Factor VM, Conner EA, Thorgeirsson SS (2011). Genomic modeling of tumor onset and progression in a mouse model of aggressive human liver cancer. *Carcinogenesis*.

[128] Fan R-H, Li J, Wu N, Chen P-S (2011). Late SV40 factor: a key mediator of Notch signaling in human hepatocarcinogenesis. *World Journal of Gastroenterology*.

[129] Qin Y, Liu J-Y, Li B, Sun Z-L, Sun Z-F (2004). Association of low p16INK4a and p15INK4b mRNAs expression with their CpG islands methylation with human hepatocellular carcinogenesis. *World Journal of Gastroenterology*.

[130] Severi T, Ying C, Vermeesch JR (2006). Hepatitis B virus replication causes oxidative stress in HepAD38 liver cells. *Molecular and Cellular Biochemistry*.

[131] Yu M-W, Yang S-Y, Pan I-J (2003). Polymorphisms in XRCC1 and glutathione S-transferase genes and hepatitis B-related hepatocellular carcinoma. *Journal of the National Cancer Institute*.

[132] Kim M, Lee HC, Tsedensodnom O (2008). Functional interaction between Wnt3 and Frizzled-7 leads to activation of the Wnt/*β*-catenin signaling pathway in hepatocellular carcinoma cells. *Journal of Hepatology*.

[133] Wieland S, Thimme R, Purcell RH, Chisari FV (2004). Genomic analysis of the host response to hepatitis B virus infection. *Proceedings of the National Academy of Sciences of the United States of America*.

[134] Gordon S (2003). Alternative activation of macrophages. *Nature Reviews Immunology*.

[135] Taylor PR, Martinez-Pomares L, Stacey M, Lin H-H, Brown GD, Gordon S (2005). Macrophage receptors and immune recognition. *Annual Review of Immunology*.

[136] Kolios G, Valatas V, Kouroumalis E (2006). Role of Kupffer cells in the pathogenesis of liver disease. *World Journal of Gastroenterology*.

[137] Bohr VA, Dianov GL (1999). Oxidative DNA damage processing in nuclear and mitochondrial DNA. *Biochimie*.

[138] Waris G, Ahsan H (2006). Reactive oxygen species: role in the development of cancer and various chronic conditions. *Journal of Carcinogenesis*.

[139] Senba M, Nakamura T, Itakura H (1985). Statistical analysis of relationship between iron accumulation and hepatitis B surface antigen. *American Journal of Clinical Pathology*.

[140] Gonzalez-Amaro R, Garcia-Monzon C, Garcia-Buey L (1994). Induction of tumor necrosis factor *α* production by human hepatocytes in chronic viral hepatitis. *Journal of Experimental Medicine*.

[141] Jia G, Takahashi R, Zhang Z, Tsuji Y, Sone H (2006). Aldo-keto reductase 1 family B7 is the gene induced in response to oxidative stress in the livers of Long-Evans Cinnamon rats. *International Journal of Oncology*.

[142] Forte TM, McCall MR, Knowles BB, Shore VG (1989). Isolation and characterization of lipoproteins produced by human hepatoma-derived cell lines other than HepG2. *The Journal of Lipid Research*.

[143] Klomp LWJ, Lin S-J, Yuan DS, Klausner RD, Culotta VC, Gitlin JD (1997). Identification and functional expression of HAH1, a novel human gene involved in copper homeostasis. *Journal of Biological Chemistry*.

[145] Liu W, Baker SS, Baker RD, Nowak NJ, Zhu L (2011). Upregulation of hemoglobin expression by oxidative stress in hepatocytes and its implication in nonalcoholic steatohepatitis. *PLoS ONE*.

[146] Boyault S, Rickman DS, De Reyniès A (2007). Transcriptome classification of HCC is related to gene alterations and to new therapeutic targets. *Hepatology*.

[147] Aravalli RN, Steer CJ, Cressman ENK (2008). Molecular mechanisms of hepatocellular carcinoma. *Hepatology*.

[148] Yuan T, Wei J, Luo J, Liu M, Deng S, Chen P (2012). Polymorphisms of base-excision repair genes hOGG1 326cys and XRCC1 280His increase hepatocellular carcinoma risk. *Digestive Diseases and Sciences*.

[149] Phillips S, Chokshi S, Riva A, Evans A, Williams R, Naoumov NV (2010). CD8+ T cell control of hepatitis B virus replication: direct comparison between cytolytic and noncytolytic functions. *Journal of Immunology*.

[150] Maini MK, Boni C, Lee CK (2000). The role of virus-specific CD8+ cells in liver damage and viral control during persistent hepatitis B virus infection. *Journal of Experimental Medicine*.

[151] Sitia G, Aiolfi R, Di Lucia P (2012). Antiplatelet therapy prevents hepatocellular carcinoma and improves survival in a mouse model of chronic hepatitis B. *Proceedings of the National Academy of Sciences of the United States of America*.

[152] Wang J, Zhao W, Cheng L (2010). CD137-mediated pathogenesis from chronic hepatitis to hepatocellular carcinoma in hepatitis B virus-transgenic mice. *Journal of Immunology*.

[153] Welzel TM, Graubard BI, Zeuzem S, El-Serag HB, Davila JA, Mcglynn KA (2011). Metabolic syndrome increases the risk of primary liver cancer in the United States: a study in the SEER-medicare database. *Hepatology*.

[154] Wong GL-H, Wong VW-S, Choi PC-L (2009). Metabolic syndrome increases the risk of liver cirrhosis in chronic hepatitis B. *Gut*.

[155] Paradis V, Zalisnski S, Chelbi E (2009). Hepatocellular carcinomas in patients with metabolic syndrome often develop without significant liverfibrosis: a pathological analysis. *Hepatology*.

[156] Cauchy F, Zalinski S, Dokmak S (2013). Surgical treatment of hepatocellular carcinoma associated with the metabolic syndrome. *British Journal of Surgery*.

[157] Turati F, Talamini R, Pelucchi C (2013). Metabolic syndrome and hepatocellular carcinoma risk. *British Journal of Cancer*.

[158] Werle-Lapostolle B, Bowden S, Locarnini S (2004). Persistence of cccDNA during the natural history of chronic hepatitis B and decline during adefovir dipivoxil therapy. *Gastroenterology*.

[159] Laras A, Koskinas J, Dimou E, Kostamena A, Hadziyannis SJ (2006). Intrahepatic levels and replicative activity of covalently closed circular hepatitis B virus DNA in chronically infected patients. *Hepatology*.

[160] Abraham TM, Loeb DD (2007). The topology of hepatitis B virus pregenomic RNA promotes its replication. *Journal of Virology*.

[161] Chong C-L, Chen M-L, Wu Y-C (2011). Dynamics of HBV cccDNA expression and transcription in different cell growth phase. *Journal of Biomedical Science*.

[162] Wursthorn K, Lutgehetmann M, Dandri M (2006). Peginterferon alpha-2b plus adefovir induce strong cccDNA decline and HBsAg reduction in patients with chronic hepatitis B. *Hepatology*.

[163] Chan HL-Y, Wong VW-S, Tse AM-L (2007). Serum hepatitis B surface antigen quantitation can reflect hepatitis B virus in the liver and predict treatment response. *Clinical Gastroenterology and Hepatology*.

[164] van Bömmel F, Zöllner B, Sarrazin C (2006). Tenofovir for patients with lamivudine-resistant hepatitis B virus (HBV) infection and high HBV DNA level during adefovir therapy. *Hepatology*.

[165] Hosaka T, Suzuki F, Kobayashi M (2010). HBcrAg is a predictor of post-treatment recurrence of hepatocellular carcinoma during antiviral therapy. *Liver International*.

[166] (2013). Liver EAftSot: novel therapeutic approaches may cure chronic HBV infection. *Novel Therapeutic Approaches May Cure Chronic HBV Infection*.

[167] Lin S-M, Lin C-J, Hsu C-W (2004). Prospective randomized controlled study of interferon-alpha in preventing hepatocellular carcinoma recurrence after medical ablation therapy for primary tumors. *Cancer*.

[168] Peters MG, Hann HW, Martin P (2004). Adefovir dipivoxil alone or in combination with lamivudine in patients with lamivudine-resistant chronic hepatitis B. *Gastroenterology*.

[169] Fung SK, Andreone P, Han SH (2005). Adefovir-resistant hepatitis B can be associated with viral rebound and hepatic decompensation. *Journal of Hepatology*.

[170] Huynh H (2010). Molecularly targeted therapy in hepatocellular carcinoma. *Biochemical Pharmacology*.

[171] Villanueva A, Llovet JM (2011). Targeted therapies for hepatocellular carcinoma. *Gastroenterology*.

[172] Frenette C, Gish R (2012). Targeted systemic therapies for hepatocellular carcinoma: clinical perspectives, challenges and implications. *World Journal of Gastroenterology*.

[173] Johnson PJ (2005). Non-surgical treatment of hepatocellular carcinoma. *HPB*.

[174] Curley SA, Stuart KE, Schwartz JM, Carithers RL (2012). *Nonsurgical Therapies for Localized Hepatocellular Carcinoma: Radiofrequency Ablation, Percutaneous Ethanol Injection, Thermal Ablation, and Cryoablation*.

[175] Curley SA, Stuart KE, Schwartz JM, Carithers RL (2013). *Nonsurgical Therapies for Localized Hepatocellular Carcinoma: Radiofrequency Ablation, Percutaneous Ethanol Injection, Thermal Ablation, and cryoablation*.

[176] Madalinski K (2008). Recent advances in hepatitis B vaccination. *Hepatitis B Annual*.

